# Global key concepts of civil-military cooperation for disaster management in the COVID-19 pandemic—A qualitative phenomenological scoping review

**DOI:** 10.3389/fpubh.2022.975667

**Published:** 2022-09-15

**Authors:** Markus Ries

**Affiliations:** ^1^Pediatric Neurology and Metabolic Medicine, Center for Pediatrics and Adolescent Medicine, University Hospital Heidelberg, Heidelberg, Germany; ^2^Center for Virtual Patients, Medical Faculty, University of Heidelberg, Heidelberg, Germany; ^3^CIMIC District Liaison Command Heidelberg, 3rd Medical Regiment, German Federal Armed Forces, Dornstadt, Germany

**Keywords:** COVID-19, pandemic, SARS-CoV-2, resilience, civil-military cooperation (CIMIC), disaster response, disaster management, disaster and emergency medicine

## Abstract

**Background:**

In the context of a holistic and comprehensive disaster response effort to the COVID-19 pandemic, many countries across the globe mobilized their military forces in order to cope with sudden and exponential surges of critically ill patients with COVID-19 in stretched healthcare systems.

**Objective:**

The purpose of this work is to identify, map, and render world-wide key concepts of civil-military cooperation (CIMIC) in disaster management during the COVID-19 crisis visible.

**Material and methods:**

Literature was systematically searched in three databases (PubMed, Web of Science, Cochrane Library) on 26 January 2022, and analyzed with qualitative, mixed narrative-phenomenological methods in compliance with PRISM-ScR and SRQR.

**Results:**

Forty-five publications were included in the analysis; pertinent authors were from 22 countries covering five continents. We identified three key thematic clusters in the published literature: Cluster (1) Medico-scientific contributions with the participation of military medical personnel or institutions: members of the military acted as subject matter experts, clinical and experimental (co-) investigators as well as co-founders for enabling COVID-19 relevant research. Areas covered were relevant to the COVID-19 patient's clinical journey from prevention, exposure, diagnostics, and treatment and included pertinent fields such as digital health and telemedicine, global and public health, critical care, emergency and disaster medicine, radiology, neurology, as well as other medical specialties, i.e., respiratory care, pulmonology, burn medicine, and transfusion medicine, in addition to environmental and occupational sciences as well as materials science. Cluster (2) CIMIC field experiences or analyses included areas such as political framework, strategy, structure, nature of civil-military interaction, and concrete mission reports in selected countries. Themes covered a broad spectrum of pandemic disaster management subjects such as capacity and surge capacity building, medical and pharmaceutical logistics, patient care under austere circumstances, SARS-CoV-2 testing support, intelligent and innovative information management, vaccination support, and disaster communication. Cluster (3) The military as a role model for crisis management.

**Conclusion:**

Civil-military cooperation made a significant contribution to the level of resilience in crisis management on a global scale, positively impacting a broad spectrum of core abilities during the COVID-19 pandemic.

## Introduction

Coronavirus disease 2019 (COVID-19) is a rapidly spreading, pandemic, multisystemic infectious disease caused by the novel coronavirus (also known as SARS-CoV-2, i.e., severe acute respiratory syndrome coronavirus 2). First cases of a “viral pneumonia” that would later be known as COVID-19 were reported in Wuhan, China, in December 2019, on 30 January the World Health Organization (WHO) declared the rapidly spreading outbreak a “Public Health Emergency of International Concern,” and finally considered the situation a pandemic on 11 March 2020 (1). By 09 June 2022, 533.766.156 cases, 6.305.234 deaths, and 11.529.693.882 SARS-CoV-2 vaccine doses administered were reported globally (2). Transmitted through aerosols from person-to-person including still asymptomatic, but yet infectious individuals, the condition spread rapidly around the globe, impacting a large geographic area in a dramatic time dynamic that was considered a VUCA (volatile, uncertain, complex, and ambiguous) public health situation for the global disaster response community (3, 4). In the context of a holistic and comprehensive disaster response effort, many countries across the globe mobilized their military forces in order to cope with sudden and exponential surges of critically ill patients with COVID-19 in stretched healthcare systems.

### Rationale for this research project

Mutual challenges within the global disaster response community were intense (5). In the author's personal experience, this led to extremely close long-term cooperation between civil and military partners, providing excellent mutual learning opportunities. In contrast, the currently available range of material in the research literature on this emerging and multi-faceted topic has not yet been well-defined in detail, which renders it difficult to obtain a clear picture on global key concepts within civil-military cooperation (CIMIC) for COVID-19 disaster relief. Strengthening global societal resilience toward crises and disaster becomes increasingly important for now and the future ahead of us. Therefore, the purpose of this work is to identify, map, and render world-wide key concepts of civil-military cooperation in disaster management during the COVID-19 crisis visible (6). We therefore focused our research efforts on the macro-level question “what was the range, extent, and nature of civil-military cooperation during the SARS-CoV-2 pandemic worldwide?,” and investigated this important issue through a broad, comprehensive scoping literature review based on three, mainly medical literature databases.

## Methods

### Quality and transparency: Research principles, methodological foundations, and guidelines

This works follows the principles and methodological framework for scoping review defined by Arksey and O'Malley, i.e., mapping range, extent, and nature of the topic investigated (6). The Preferred Reporting Items for Systematic Reviews and Meta-Analyses extension for Scoping Reviews (PRISMA-ScR) checklist was applied for design, execution, analysis, and reporting (7). The qualitative aspects of this work follow the Standards for Reporting Qualitative Research (SRQR) checklist (8). A review protocol was not registered.

### Information sources, search strategy, and eligibility

We searched three literature databases on 26 January 2022: PubMed (9), Web of Science (10), and Cochrane Library (11). A combination of three databases was chosen, because the literature included in each individual database has a specific focus; the goal of the combination was to cover a broader spectrum. COVID-19 is mainly a medical issue, therefore the three databases were chosen, because they index predominantly medical literature in contrast to other databases that focus more on sociological research.

The search parameters in the advanced search functions in all three databases were: “civil” and “military” and “COVID” as well as “CIMIC” (which is the abbreviation for “civil-military cooperation”) and “COVID.” No filter was applied. The search was non-iterative and was not modified over time.

Literature search results were transferred to the citation manager Zotero (12). Duplicates were removed. Articles were retrieved and screened. Publications were considered eligible if their focus was centered on any aspect of civil-military cooperation in the COVID-19 pandemic. Articled not fulfilling this broad eligibility criterion were excluded during screening. In order to enhance trustworthiness, a critical appraisal of credibility was undertaken based on the professional judgment of the author. Languages considered a priori were English, German, French, and Spanish as the author is fluent in these. All levels of evidence were considered.

### Data charting: Qualitative literature analysis, coding of significant statements and horizontalization in order to identify global CIMIC key concepts

Identified publications were analyzed through a qualitative, mixed narrative-phenomenological approach as proposed by Creswell, and as applied in previous projects of similar scope published in the peer-reviewed literature (3, 4, 13, 14). This approach was considered best for providing the highest flexibility for identification of pertinent key topics rather than analyzing data automated with pre-specified terms which would potentially miss significant statements. Specifically, narrative-phenomenological qualitative information in the articles was identified by transcribing articles into a plain text format that were assessed for significant statements as the narrow unit of analysis (13, 14). These significant statements were then horizontalized and grouped into clusters of meaning (13, 14). The coding was conducted with the open source qualitative data analysis package RQDA 0.2–8 (15) in R (16) operated with Linux Mint 20 (17).

### Data items and analysis

The following variables were qualitatively analyzed for significant statements, themes and clusters of meaning: title of publication, main subject, geographical focus, time (of the pandemic the report refers to), type of medical contribution of the military, medical specialty field of the overall work, methodology of the scientific work, and country of first author (for analytical perspective) or military (co-) author(s) (for medico-scientific context). For military context, the North Atlantic Treaty Organization (NATO) membership (for military context), membership of European Union (for political context), and continent (for wider geographical context) were considered contextual variables for country of first author. Continents were designated according to the nomenclature provided by the U.S. Geological Survey (18). In order to gain a visual insight into the literature's global distribution patterns, countries of first authors' or military (co-) authors affiliations were mapped with QGIS 3.10.4 (LTR)-A Coruña for Linux Ubuntu using Natural Earth vector map data (19, 20). For medico-scientific articles, military authors' affiliation countries were mapped in order to show where the *military* contributions- which were often large multinational multicenter studies—originated from. For CIMIC field experiences or analyses, the country of the first author of the publication identified was mapped, because the intention was to focus on the *contextual national perspective* of the contribution to the literature. The thematic mind map and bubble graph were drawn with MindMaster (21).

### Researcher characteristics and reflexivity

Qualitative data analysis can be influenced by the researcher's characteristics and reflexivity. The investigator of this work is a pediatric clinician-scientist practicing, researching, and teaching at the University Hospital Heidelberg, Germany, and the University of Heidelberg, Germany, with training or work experience in Germany, the United States, France, Spain, the UK, Chile, and at the NATO (North Atlantic Treaty Organization). He serves as a reserve medical staff officer in the rank of a Lt.-Colonel (OF-4) of the German Armed Forces in the local CIMIC command. Since March 2020, he has been involved directly in coordinating military-medical COVID-19 disaster management support for the city and healthcare system of Heidelberg, Germany, as well as for hospitals in the surrounding region.

## Results

**Figure 1:**
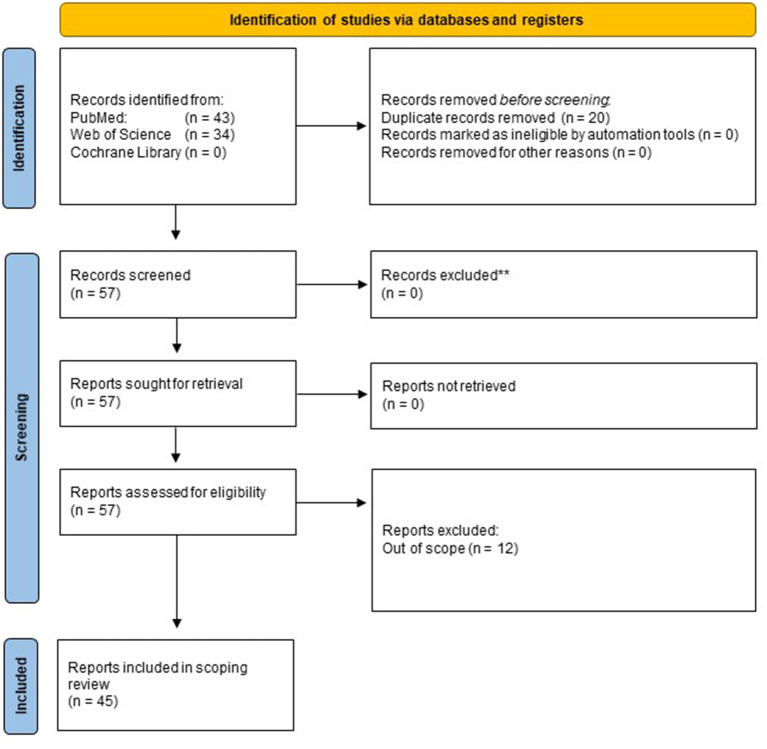
Literature search strategy PRISM flow chart. Three databases (PubMed, Web of Science, and Cochrane Library) were considered. Close of database was 26 January 2022.

After a careful identification and screening process as illustrated in the PRISMA flow diagram (Figure 1), forty-five publications were included in the analysis. Articles were published in English, French, or Spanish.

Three thematic clusters were identified in the phenomenological analysis (Table 1).

**Table 1:** Key concepts of civil-military interaction for disaster management during the COVID-19 pandemic: phenomenological clusters of meaning in the medical literature (searched in PubMed. Web of Science, Cochrane Library, close of database 26 January 2022).

**Cluster No**.	**Cluster theme**	**Number (%) of publications** **[Σ = 45]**
1	Medico-scientific contributions with the participation of military medical personnel or institutions	*N =* 24 (53%)
2	CIMIC field experiences or analyses	*N =* 18 (40%)
3	The military as a role model for crisis management	*N =* 3 (7%)

Reports covered five continents: Asia, Europe, North America, South America, Oceania. First authors of CIMIC field reports, role model reports, and (co-) authors for medical studies included 22 different countries. Further details on thematic and geographic distribution will be presented and discussed below.

### Medico-scientific contributions with the participation of military medical personnel or institutions

**Figure 2:**
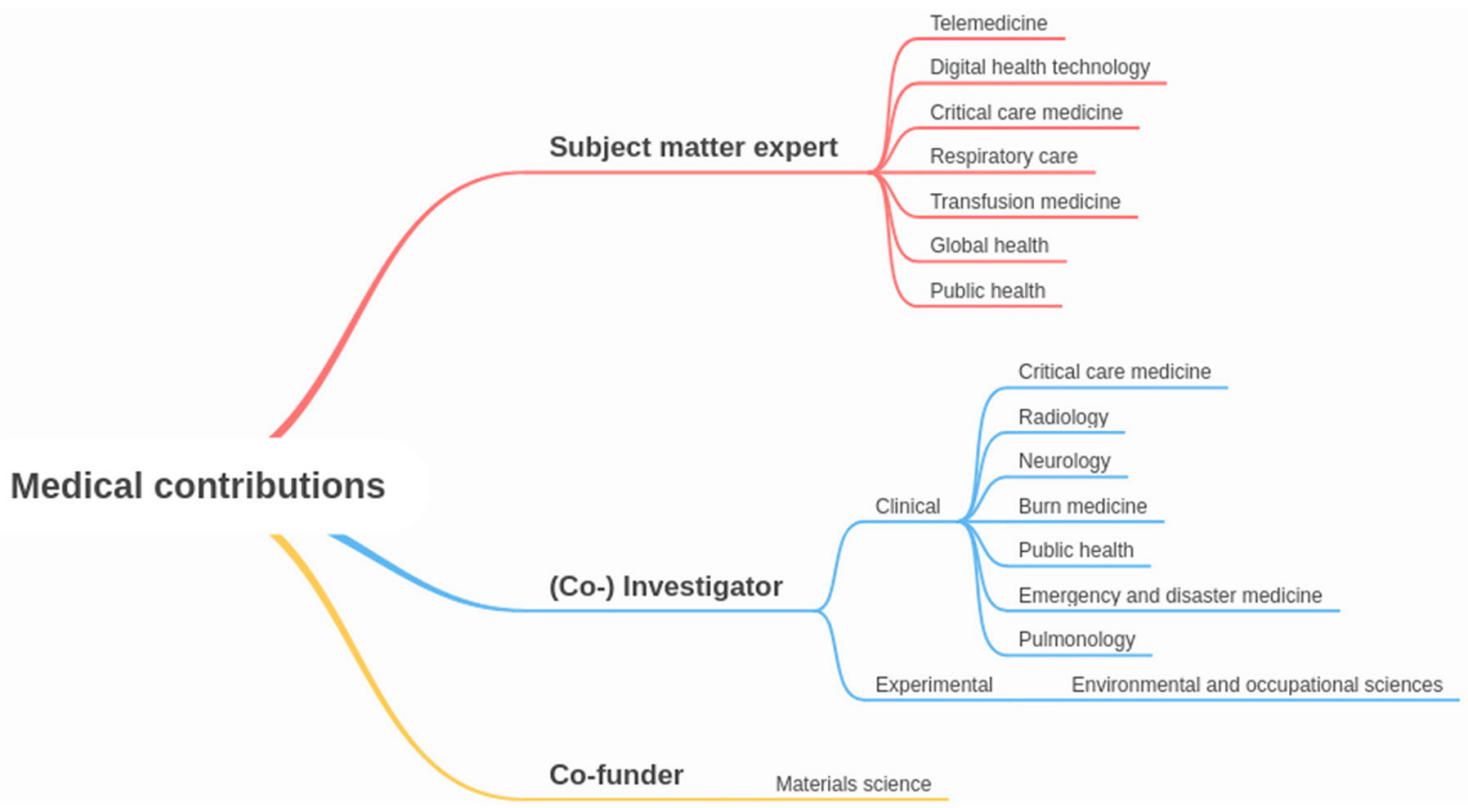
Mindmap of medical contributions with the participation of military medical personnel or institutions by role and specialty area covered.

For medico-scientific contributions to the literature within CIMIC, the military acted in three distinct roles: (1) as subject matter experts, (2) as clinical or experimental investigators or co-investigators, and (3) as co-funders for research (22–45). Military subject matter expertise included telemedicine, digital health technology, critical care medicine, respiratory care, transfusion medicine, as well as global and public health. Military clinical investigators or co-investigators contributed to COVID-19 relevant topics such as critical care medicine, radiology, neurology, burn medicine, public health, emergency and disaster medicine, and pulmonology. One experimental military investigator worked within the field of environmental and occupational sciences. A synoptic overview of each contribution is provided in Figure 2, detailed information about each publication is listed in Table 2.

**Table 2:** Synopsis of medical publications that included the participation of military medical personal or institutions.

**No**.	**Title of publication**	**Type of medical contribution of the military**	**Medical specialty**	**Methodology**	**Country of military (co-) author(s)**	**Reference**
1	Advanced digital health technologies for COVID-19 and future emergencies	Subject matter expertise	Telemedicine and digital health technology	Expert review of medical literature and science & technology news	USA	(22)
2	Awake prone positioning in non-intubated patients with acute hypoxemic respiratory failure due to COVID-19	Subject matter expertise	Respiratory care / Critical care medicine	Meta-analysis of published observational studies	China	(23)
3	Civilian walking blood bank emergency preparedness plan	Subject matter expertise	Transfusion medicine	Expert panel establishing a planning for transfusion of emergency untested whole blood in situations of donor shortage	Norway, USA	(24)
4	Classroom aerosol dispersion: desk spacing and divider impacts	Experimental investigator	Environmental and occupational sciences	Classroom aerosol dispersion study	USA	(25)
5	Collective aeromedical transport of COVID-19 critically ill patients in Europe: A retrospective study	Clinical investigator	Critical care medicine	Retrospective analysis of clinical data	France	(26)
6	Ebola, COVID-19 and Africa: What we expected and what we got	Subject matter expertise	Global health	Country report on the public health situation in the Democratic Republic of the Congo in the context of a peacekeeping mission	India	(27)
7	Efficacy of Chest CT for COVID-19 Pneumonia Diagnosis in France	Clinical Co-investigator	Radiology	Diagnostic survey study	France	(28)
8	French multicentre observational study on SARS-CoV-2 infections intensive care initial management: the FRENCH CORONA study	Clinical Co-investigator	Critical care medicine	Observational study on clinical management	France	(29)
9	Global Health Security Alliance (GloHSA)	Subject matter expertise	Public Health	Proposal for a common European pandemic crisis and disaster management mechanism which includes NATO capabilities	Germany	(30)
10	Global impact of COVID-19 on stroke care and IV thrombolysis	Clinical Co-investigator	Neurology	Observational study on the effect of COVID-19 on stroke hospitalizations and interventions	China, Czech Republic, Poland, Tunisia	(31)
11	Impact of COVID-19 on global burn care	Clinical Co-investigator	Burn Medicine	Observational study on the effect of COVID-19 on burn care, management, and resources	China, Japan	(32)
12	Implementing public health strategies-the need for educational initiatives: a systematic review	Subject matter expertise	Public Health	Literature review of public health education strategies	Poland, Sweden	(33)
13	Incidence of SARS-CoV-2/COVID-19 in military personnel of Bolivia	Clinical Co-Investigator	Public Health	Description of the SARS-CoV-2 epidemiological situation in Bolivia	Bolivia	(34)
14	Mass-surveillance technologies to fight coronavirus spread: the case of Israel	Subject matter expertise	Public Health	Lessons learned from the Israeli experience with cellphone tracking for epidemiological surveillance	Israel, USA	(35)
15	Modeling of Various Spatial Patterns of SARS-CoV-2: The Case of Germany	Investigator	Public Health	Modeling SARS-CoV-2 outbreaks in Germany	Poland	(36)
16	Moving forward from COVID-19: Organizational dimensions of effective hospital emergency management	Co-investigator	Emergency and Disaster Medicine	Assessment of preparedness of US hospitals for emergencies and proposal for a culture in supporting effective emergency management	USA	(37)
17	Multi-agent simulation model for the evaluation of COVID-19 transmission	Investigator	Public Health	Development of a multimodal model that simulates SARS-CoV-2 transmissions in the population	Brazil	(38)
18	Outcomes of COVID-19-related ARDS patients hospitalized in a military field intensive care unit	Clinical investigator	Critical care medicine, Disaster Medicine	Observational study on clinical management of COVID-19 patients in a field hospital	France	(39)
19	Personal view: security sector health systems and global health	Subject matter expertise	Public Health, Disaster Medicine	Analysis of the contribution of the security sector health system to government health services and crisis response	UK	(40)
20	Pulmonary embolism and deep vein thrombosis in COVID-19: a systematic review and meta-analysis	Subject matter expertise	Radiology	Meta-analysis of a systematic literature review	France	(41)
21	Reduced maximal aerobic capacity after COVID-19 in young adult recruits, Switzerland, May 2020	Clinical Investigator	Pulmonology	Controlled cohort study comparing physical endurance before and after a COVID-19 infection	Switzerland	(42)
22	Social distancing alters the clinical course of COVID-19 in young adults: a comparative cohort study	Clinical investigator	Public Health	Controlled cohort study comparing SARS CoV-2-infection rates before and after implementation of social distancing	Switzerland	(43)
23	The difficulties, opportunities and challenges of COVID-19 by Chinese medicine on China	Subject matter expertise	Public Health	Review of the Chinese COVID-19 management strategy	China	(44)
24	Visible-light-driven and self-hydrogen-donated nanofibers enable rapid-deployable antimicrobial bioprotection	Civil-military co-funding	Material Sciences	Development of antiviral nanofibers	China	(45)

Articles are listed in alphabetical order. The literature search was performed using Pubmed. Web of Science, and Cochrane Library, close of database was 26 January 2022.

**Figure 3:**
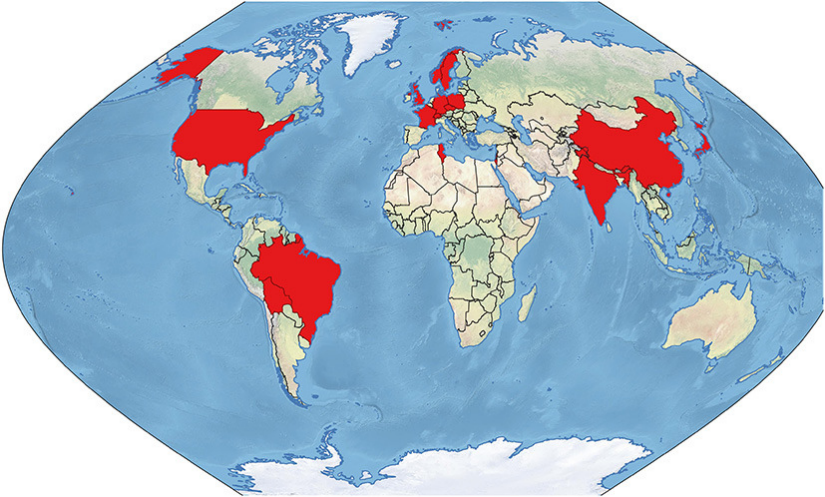
Countries of military (co-) authors' affiliation in medico-scientific civil-military publications during the COVID-19 pandemic (in red).

The map in Figure 3 shows countries of military (co-) authors in medico-scientific publications with military contributions during the COVID-19 pandemic.

### CIMIC field experiences or civil-military analyses

**Figure 4:**
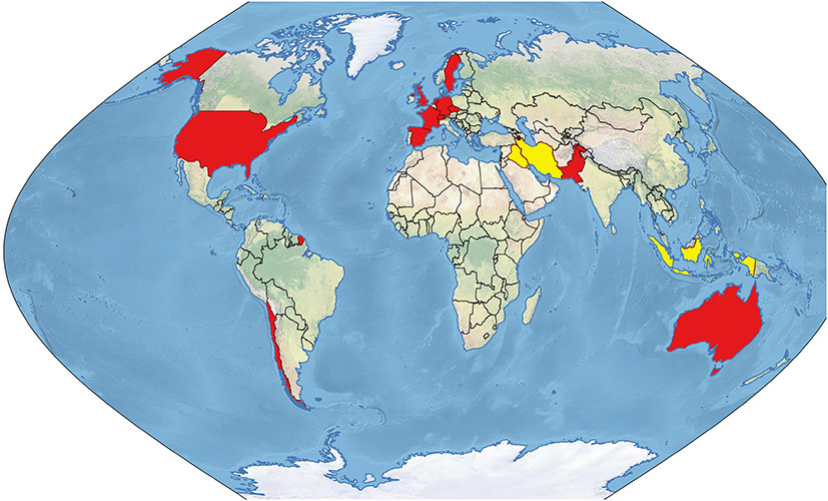
CIMIC field experiences and analyses during the COVID-19 pandemic. Red: first authors' countries of affiliation. Yellow: Countries in which CIMIC activities were analyzed by authors from other countries.

The following section reviews concrete CIMIC field experiences or analyses of such (3, 46–62). Because disaster management during COVID-19 was generally guided by national frameworks and policies, pertinent reports in this section are reviewed grouped by country of first author in order to maintain this perspective (Figure 4), appearing geographically aggregated by continent and in descending order of overall numbers of reports per country. Further details, including time periods of reports covered, are provided in Table 3.

**Table 3:** Synopsis of CIMIC experiences reported in the global academic literature.

**No**.	**Continent**	**Perspective (country of first author)**	**Main subject**	**Geographical focus**	**Time**	**Reference**
1	EU	UK^¶^	Themes (seven identified) and intensity of CIMIC support (highest in Spain and Italy, lowest in Sweden)	UK, France, Spain, Italy, Belgium and Sweden	20 January to 21 March 2020	(46)
2	EU	UK^¶^	Three emerging trends of national military involvement: minimal technical military support, blended civil-military responses, military-led responses	Global	Initial stage of the COVID-19 pandemic	(47)
3	EU	UK^¶^	Disaster response capacity building in the public service	UK	Prior to the COVID-19 pandemic	(48)
4	EU	UK^¶^	Assistance in transport of ventilated patients during a COVID-19 surge and lessons identified	UK (London)	14 to 26 April 2020	(49)
5	EU	Germany^¶^^*^	Development of innovative disaster management tools: information management, crisis communication, data-visualization, training of supporting staff, framework and evaluation concept	Germany	1^st^ and 2^nd^ wave of the COVID-19 pandemic in Germany	(50)
6	EU	Germany^¶^^*^	Emergency roll-out of SARS-CoV-2 vaccination campaign: strategic elements, planning and analysis tools, lessons learned, top ten priorities and pitfalls	Germany	November 2020 to April 2021	(3)
7	EU	France^¶^^*^	Broad military support of civilian partners, adaptation of efforts to local needs, dialogue is important	France	Starting 25 March 2020	(51)
8	EU	The Netherlands^¶^^*^	Deployment of armed forces were leveraged by political framing of the pandemic as war. Operational readiness and societal standing of militaries improved, with necessary emphasis on civilian control and civil rights	Global	COVID-19 pandemic in general	(52)
9	EU	Czech Republic^¶^^*^	Development of a (largely mathematical) method to (1) determine the degree of preparedness of the Czech army for cooperation with civilian partners in in disaster management including COVID-19 and (2) to identify areas for improvement	Czech Republic	COVID-19 pandemic and disasters in general	(53)
10	EU	Spain^¶^^*^	Crisis communication of the government was centered around four axes: (1) continuous communication, (2) seriousness of the crisis, (3) feeling of control, (4) unity. Prominent visibility of military in the public communication visibility due to its high esteem in the population	Spain	15 March to 25 April 2020	(54)
11	EU	Sweden^*^	Preparedness ahead of the curve: Deployment of a mobile laboratory and PCR tests to diagnose SARS-CoV-2 infections	Sweden	End of January 2020	(55)
12	EU	Switzerland	Enlistment of civilian pharmacists in the Medical Service of the Swiss Armed Forces as militia officers and their roles in hospital battalions and a medical logistic battalion	Switzerland	6 March to 30 June 2020	(56)
13	NA	USA^¶^	Deployment of 500 Navy Reserve medical professionals to New York City, supporting in part eleven overburdened local hospitals	USA	April to June 2020	(57)
14	NA	USA^¶^	Civil-military cooperation by rapid activation and operation of a COVID-19 inpatient care facility in a congress center in New York City (Army medical service)	USA	20 March to 1 May 2020	(58)
15	SA	Chile	Military deployed in the streets during nightly curfew	Chile	Based on decrees of March 22 and 4 August 2020	(59)
16	OC/AS	Australia	Indonesian armed forces and state intelligence service were given prominent roles in in the production of anti-COVID-19 medicine and COVID-19 testing, and enforcement of pandemic mandated restrictions in the society which contributed to weakening democracy	Indonesia	After March 2020	(60)
17	AS	US	Iraqi paramilitary units and militia transported medical supplies, personal protective equipment, food, sanitized public spaces, conducted medical information campaigns, provided mental health support to medical personnel, buried the deceased respecting religious rituals (including Muslim and Christian faith), constructed field hospitals (including a 200-bed hospital in Baghdad) Revolutionary Guards and militia in Iran built field hospitals and enforced quarantine	Iraq and Iran	COVID-19 pandemic in general	(61)
18	AS	Pakistan	Four themes: (1) significance of CIMIC in disaster management, (2) challenges during the COVID-19 pandemic (3) role of a common civil-military comment operation center (4) government policies and practices related to disaster management	Pakistan	April 2020 to September 2020	(62)

The literature search was performed in PubMed. Web of Science, and Cochrane Library, close of database was 26 January 2022.

¶ Denotes countries with NATO membership.

* Denotes countries with European Union membership.

Continents: AS, Asia; EU, Europe; NA, North America; SA, South America; OC, Oceania (18).

#### United Kingdom

Gad et al. analyzed civil-military cooperation in six European countries, i.e., UK, France, Spain, Italy, Belgium and Sweden, in the early phase of the COVID-19 crisis (46). For this analysis, they identified seven main analytical themes, i.e., (1) Recognition of health security threat from coronavirus spread in Wuhan, (2) detection and announcement of first cases as reported through military health functions, (3) invocation or announcement of national crisis, plans and/or military involvement, (4) how military support was incorporated into national crisis response, (5) how the military modified its activities, (6) dealing with rumors/allegations related to COVID-19, and (7) other—military and COVID-19, and divided these themes into 19 categories of civil-military cooperation (46). The armed forces and the military medical service were key components of early disaster response and strengthened resilience, while Italy and Spain had the most intense and Sweden the least intense level of CIMIC within this group of countries (46). Gibson-Fall identified three different trends of national military involvement during the COVID-19 crisis worldwide: (1) minimal technical military support, (2) blended civil-military responses, and (3) military-led responses (47). An interesting example for enhancing crisis management capabilities in the public sector is the British stabilization unit, which facilitates cooperation between agencies, civilians and the military and could serve as a training and capacity building model (48). Ten military critical care transfer teams assisted the London Ambulance Service and transported 52 ventilated civilian patients during a COVID-19 patient surge in intensive care units in London, UK, during the last 2 weeks in April 2020 (49). Each two-member team was composed of (1) a consultant/registrar in emergency medicine and pre-hospital emergency medicine or anesthesia and (2) an emergency nurse or paramedic (49). Main lessons identified centered around overcoming technical issues with the ventilation and measures to avoid transmission SARS-CoV-2 to the staff (49).

#### Germany

Roßmann et al. focused on systems innovation, analyzing the dynamic challenges of the emerging COVID-19 pandemic through a Cynefin lens; very similar crisis management problems were found in different areas of the public health service in Germany (50). They identified four key areas that necessitated systems innovation to strengthen disaster resilience, i.e., (1) information-management including crisis communication, (2) data- and information-visualization (dashboard), (3) training and education of supporting staff, and (4) a framework and evaluation concept (“scoring-matrix”), and developed novel tools to adapt, change, and innovate the public disaster management system (38).

Schulze et al. described lessons learned during the SARS-CoV-2 emergency vaccination roll-out campaign in Heidelberg in the year 2020. The following five strategic elements were important for success: (1) robust mandate, (2) use of established networks, (3) fast on-boarding and securing of commitment of project partners, (4) informed planning of supply capacity, and (5) securing the availability of critical items (3). Planning tools included (1) analyses through a VUCA lens, (2) analyses of stakeholders and their management, (4) possible failures, and (5) management of main risks including mitigation strategies (3). Lessons learned identified ten tactical leadership priorities and ten major pitfalls. The authors proposed that these methods which comprised VUCA factors combined with analyses of possible failures, and management of stakeholders and risks could be adjusted to any public health care emergency anywhere across the globe in the future (3).

#### France

Barreau summarized the French civil-military operation which was entitled “resilience” and launched on 25 March 2020. The military adapted their support to the local needs and circumstances, a permanent dialogue between the civilian and military partners was important (51).

#### The Netherlands

In a global analysis of military deployments in the COVID-19 crisis, Kalkman described circumstances, motivations and societal imitations of these endeavors. As such, the political framing of the pandemic as a “war,” e.g., in the US, in France, and in the UK, leveraged and triggered a military response as a logical consequence out of this narrative (52). Moreover, these deployments were also in the interest of the militaries, because they strengthened their operational readiness and societal standing as they assisted the population (52). Of note, Kalkman emphasized the necessity of civilian control and respect of civil rights for reasons of a cooperative leadership culture and balanced disaster management approach (52).

#### Czech Republic

Assessment of military preparedness for civil-military cooperation in a disaster situation can be challenging and complex. Therefore, Tušer and colleagues developed a capacity and capability assessment procedure based on questionnaires and a mathematical model which includes Saaty's method (53). The goal was to determine the degree of preparedness of the Czech army for cooperation with civilian partners in disaster management including the COVID-19 crisis. and to identify specific areas for improvement (53). The four assessment criteria included (1) human resources, (2) technical security of allocated forces, (3) command and control of allocated forces, and (4) planning; these criteria were further subdivided into two or three indicators each (53).

#### Spain

Consistent with the report by Gad, Lopez-Garcia observed a high degree of visibility of the military and other security institutions in the crisis communication strategy of the Spanish government (46, 54). The four key axes of the crisis communication in Spain were (1) continuous communication, (2) seriousness of the crisis, (3) feeling of control, and (4) unity (54). This highly visible presence was a result of the high degree of trust that the military was enjoying in Spain compared with other public, political, private, and religious institutions. Thus, an association with the military during the COVID-19 crisis had a protective function for Spanish politicians against critics from the opposition (54).

#### Sweden

Bacchus and colleagues emphasized the necessity of thorough inter-agency preparedness for disasters in advance (55). The report civil-military experience with the rapid deployment—initially a high readiness exercise in January 2020—of a military mobile biological field analysis laboratory and the development of a polymerase chain reaction (PCR) test in order to facilitate the diagnosis of SARS-CoV-2 infections (55). This project was a collaboration of the Swedish Armed Forces, the Public Health Agency, and a civilian hospital (55).

#### Switzerland

In Switzerland, civilian pharmacists were enlisted as reserve officers in the military and supported the civil-military crisis response in hospital battalions and medical logistics battalions (56). Overall, 5,000 mostly medical soldiers including pharmacists were mobilized 6 March to 30 June 2020 within the Swiss militia system (56). In the hospital battalion, they mainly managed supply of medical material to military and civilian entities and coordinated hygiene measures to reduce the risk of staff contamination with SARS-CoV-2 (56). Their main duty in the medical logistics battalion included pharmaceutical production support in civilian and military facilities (56).

#### USA

There were two remarkable project reports on civil-military cooperation from the US. First, Dutta et al. described the deployment of 500 Navy Reserve medical professionals to New York City (57). Some of these reservists supported eleven local hospitals that were overburdened with the COVID-19 surge which led to the exhaustion of the civilian staff. This civil-military mission was an example for successful rapid deployment of medical forces and cohesive cooperation in a diverse professional setting across all specialties (57). Likewise, the Army medical service supported New York City as well. They rapidly activated and operationalized a COVID-19 inpatient care facility in a civilian congress center in New York City, successfully integrating uniformed services, governmental agencies, and private healthcare organizations (58).

#### Chile

In the context of the socioeconomic tensions, the military was deployed in the streets during nightly curfews based on two government decrees in Chile, reported Dragnic, a sociologist at the University of Chile (59).

#### Indonesia (From an Australian perspective)

Fealy, a scholar from The Australian National University published a critical but important analysis of the role of the Indonesian armed forces in the COVID-19 crisis. The Indonesian armed forces and state intelligence service had very prominent roles during the pandemic—which he considered disproportionate—that resulted in weakening democracy in Indonesia (60). Specifically, despite lacking expertise, they were involved in the production of anti-COVID-19 medicine and COVID-19 testing (60). Furthermore, the military was tasked with the enforcement of restrictions mandated by the spread of the virus in the society and were given the authority to impose punishment on citizens (60).

#### Iran (From a U.S. perspective)

In Iran, Revolutionary Guards and the affiliated militia supported the COVID-19 disaster response by building field hospitals and enforcing quarantine (61).

#### Iraq (from a U.S. perspective)

Of interest, in Iraq, paramilitary forces and militia took over roles and responsibilities that one would expect being led and fulfilled by the government as well as the public health sector. Specifically, Iraqi paramilitary units and militia contributed to mitigating the impact of the pandemic by providing logistic support, i.e., transporting medical supplies, personal protective equipment, and food (61). They supported hygiene measures by sanitizing public spaces, but also covered typical public health activities such as medical information campaigns (61). Their approach appeared to be comprehensive and covered mental health support to medical personnel and the construction of field hospitals including a 200-bed hospital in Baghdad (61). In addition, these groups helped burying the deceased while respecting diverse religious rituals including both Muslim and Christian faith (61).

#### Pakistan

Jabbar and Makki analyzed civil-military cooperation during the COVID-19 pandemic from a leadership perspective (62). They focused on four themes, i.e., (1) the significance of CIMIC in disaster management, (2) challenges associated with CIMIC during the COVID-19 pandemic, (3) the role of a common civil-military comment operation center, and (4) government policies and practices related to disaster management (62). Of interest, most funding is spent into measures responding to a disaster rather than in prevention (62). This is not an isolated phenomenon, but a frequent global shortcoming, which is being addressed by the Sendai Framework for Disaster Risk Reduction 2015–2030 (63). Tasks of the Pakistani army included support in SARS-CoV-2-testing, logistics (i.e., distribution of medical equipment including testing kits, ventilators, personal protective equipment and drugs), disaster response coordination, and information management (62). In Pakistan, overcoming a communication gap and fostering collaboration between civilian actors was important during the COVID-19 crises (62). In the absence of international guidelines for CIMIC, a definition of the overall framework and guidelines are helpful (62). Joint training of civilian and military stakeholders improved functioning and mutual understanding, and increases trust, while previous military training of civilian actors proved to be beneficial in this regard (62).

### The military as a role model for crisis management

In a New England Journal of Medicine editorial, Michael emphasized a tradition of influence from of military medicine on to its civilian partners (64). Two recent examples in the COVID-19 pandemic corroborated this relationship and dialogue. Successful management of SARS-CoV-2 outbreaks on a ship and in a Marines boot camp delivered valuable insight into virus transmission, disease understanding, diagnosis, tracking and tracing as well as appropriate quarantine measures in the early phase of the pandemic that could be extrapolated into civilian community settings such as schools, dorms, or other shared living environments (64). Katz and colleagues considered items of military medicine such as preparedness, team-based care, echelons of care, augmenting the effort, effective triage, and servant leadership as important lessons learned for adaptation into cardiac critical care during the COVID-19 pandemic (65). While hierarchical top-down, command-and control structures in healthcare may have worked well in the past in military operations, crisis management, and certain healthcare settings, they do not meet today's standards due to generational value change and complexity issues in the operational environment (66). There has been a slow shift in healthcare leadership culture toward the emphasis on emotional intelligence in order to (1) foster respect and civility to empower teams, (2) lead with transparency and open communication to promote psychological safety, and (3) lead with compassion when tackling severe problems. This change process may now experience push-backs and regression into the old-school system because of the pressure during the pandemic (66). Role model articles originated from the U.S. and Canada.

## Discussion

The purpose of this work was to identify, map, and render world-wide key concepts of civil-military cooperation in disaster management during the COVID-19 crisis visible. This scoping review of identified three key thematic clusters in the published literature: (1) Medico-scientific contributions with the participation of military medical personnel or institutions (2) CIMIC field experiences or analyses and (3) the military as a role model for crisis management. Pertinent authors were from 22 countries covering five continents.

For medico-scientific contributions, members of the military acted as subject matter experts, clinical and experimental (co-) investigators as well as co-founders for enabling COVID-19 relevant research. Areas covered were relevant to the COVID-19 patient's clinical journey from prevention, exposure, diagnostics, and treatment and included pertinent fields such as digital health and telemedicine, global and public health, critical care, emergency and disaster medicine, radiology, neurology, as well as other medical specialties, i.e., respiratory care, pulmonology, burn medicine, and transfusion medicine. Environmental and occupational sciences as well as materials science were represented, too.

**Figure 5:**
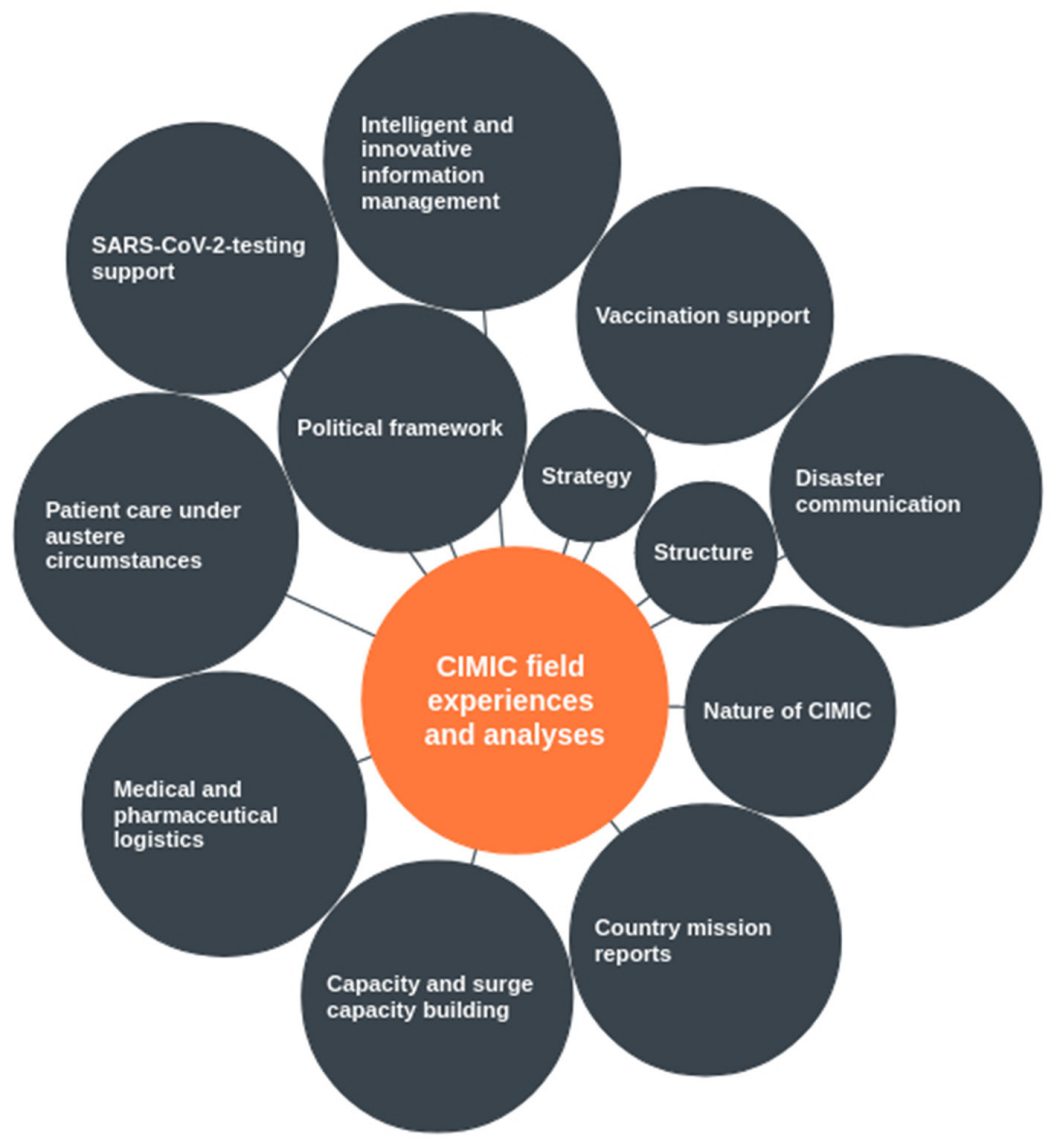
Themes covered in CIMIC field experiences and analyses. Within individual reports, themes overlapped in part.

CIMIC field experiences and analysis included areas such as political framework, strategy, structure, nature of civil-military interaction and concrete mission reports in selected countries. Themes covered a broad spectrum of pandemic disaster management subjects such as capacity and surge capacity building, medical and pharmaceutical logistics, patient care under austere circumstances, SARS-CoV-2 testing support, intelligent and innovative information management, vaccination support, and disaster communication (Figure 5).

Finally, specific aspects of leadership, training, and operational capacities within the military were considered helpful and could provide role models in certain civilian emergency and disaster situations, recognizing at the same time that leadership cultures are subject to particular circumstances and change over time.

The vast majority of articles the role of the military during the COVID-19 pandemic was reported in a neutral or positive perspective in the literature reviewed here. Positive experiences included the military contribution to the advancement of medical and scientific knowledge, and providing medical care in austere circumstances. Furthermore, the use particular, sometimes even unique capabilities of the military such as leadership, technical, logistical, and organizational skills, innovative thinking, as well as the availability of rapidly deployable manpower and equipment for the purpose of serving the population and resulting in tangible disaster relief were positive, and well-received examples that should be followed in the future. On the contrary, the political frameworks of civil-military cooperation in particular in Indonesia and Chile were discussed critically by other authors (59, 60). Likewise, Medeiros Passos and Acácio analyzed the impact of military involvement in Latin American countries, in particular policing missions in the Dominican Republic, Ecuador, El Salvador, Guatemala, Bolivia, Honduras, Chile, as well as Peru, and addressed the important issue of short term-human rights degradation and aggravation of police violence (67). Furthermore, they concluded that the attribution of disaster management positions to military personnel in Brazil, Chile, Bolivia, and Peru may have weakened the civilian control of the armed forces in the future (67).

When thinking about disaster management in general, it is of utmost importance to have a fundamental understanding about the basic needs of the potentially afflicted population including the vulnerable (68, 69). For a general assessment of civil-military cooperation and a complementary regard on Gibson-Fall's valuable analysis of the three different trends of national military involvement during the COVID-19 pandemic (47), the humanitarian perspective of the United Nations is very interesting in terms of workload sharing and degree of military visibility. The UN cluster approach provides a helpful overview and orientation for main sectors of humanitarian action and disaster relief. The following functional areas are considered: camp management, early recovery, children and education, emergency telecommunications, food security, health, logistics, nutrition, protection, shelter, WaSH (water, sanitation, hygiene) (70). In principle, the UN recognizes that the military's specific capabilities and capacities are a valuable asset in humanitarian actions which includes disaster management (71). When wanting to work successfully together with a non-governmental or humanitarian organization in a disaster relief mission, it is of specific importance to both medical and non-medical military CIMIC officers to take into account the humanitarian principles—humanity, neutrality, impartiality, and operational independence—that guide these organizations, because they provide the fundamental principles for their members' cultural and social mindsets (71, 72). From a UN perspective, the framework for CIMIC-relationships between the military and civilian humanitarian organizations is determined by two key considerations: is the situation (1) a natural, technological, or environmental emergency in times of peace assuming a stable government and the state providing for security, or (2) is the emergency situation complex, i.e., military and other armed actors are or are perceived as party to the conflict and thus humanitarian actors would avoid any association with military actors and minimize their interaction (72). In peacetime, humanitarian actors would seek a *cooperative* approach to civil-military interaction, whereas in complex emergency situation with direct involvement of the military in the conflict, they would rather choose a *co-existent*, i.e., a more distant and indirect, less visible relationship with military actors in disaster relief (72). Therefore, in humanitarian actions or disaster relief situations, the United Nations distinguish three types of military assistance with decreasing visibility to the public: (1) direct assistance, (2) indirect assistance, or (3) infrastructure support. This graduated visibility concept is illustrated in Table 4 which provides an overview about these three concepts and further details and examples. As COVID-19 crisis management occurred in general in a peace time setting, direct civilian-military information exchange and even direct military assistance to the population—within the constitutional framework under civilian leadership—would not pose any issue to civilian organizations including NGOs or other humanitarian actors. In any case, a close dialogue between military and civilian or humanitarian actors is considered essential and key elements of humanitarian civil-military interaction includes information sharing, task division and joint planning as well as practical partnership and operative engagement (71, 74).

**Table 4:** Types of military assistance in humanitarian or disaster situations according to the United Nations Civil-Military-Coordination Field Handbook (72).

**Symbol**		**Type of assistance**	**Definition (72)**
“The cookie”	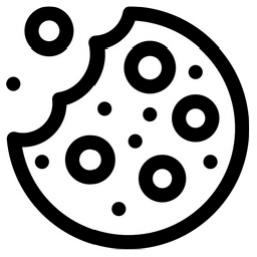	Direct assistance	“Face-to-face distribution of goods and services”
“The truck”	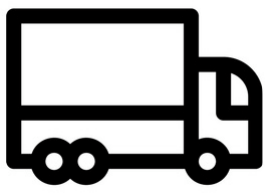	Indirect assistance	“At least one step removed from the population – transporting relief goods, building camps and shelters, providing water sources, clearing mines and ordnance, etc.”
“The bridge”	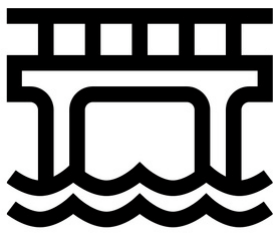	Infrastructure support	“General services that facilitate relief, but are not necessarily visible to, or solely for, the benefit of the affected population - repairing infrastructure, operating airfields, providing weather info, ensuring access to communications networks, etc.”

Image credit: this table was designed using freely available resources from Flaticon (73).

### Limitations

This work has several limitations that have to be taken into account for the appropriate interpretation of this scoping review. In order to avoid selection bias, a systematic and transparent literature search, screening and inclusion was conducted. This work probably under-reports the full extent of civil-military cooperation during the COVID-19 pandemic, because it is very likely that not all CIMIC experiences in the field were published in the literature. The inclusion of other databases or even gray literature may have resulted in further nuances. The qualitative analysis within the present work was—as in any qualitative study—subjected to the characteristics and reflexivity of the researcher, therefore, subjectivity bias cannot be excluded. Nevertheless, this report covers information from established, robust and credible medical and scientific databases which might contribute to the specificity of this scoping review. We consider this scoping review informative, because common global themes of the pandemic were identified, and we consider these data generalizable within the context of the above-described, important limitations.

## Conclusion and directions for future research

Data in this scoping review suggest, in general, that civil-military cooperation substantially contributed to societal resilience in crisis management on a global scale in a broad spectrum of core abilities during the COVID-19 pandemic—presumably at a high cost. If the health care system in a particular country is overstretched before a crisis occurs, the mitigation potential be limited whoever the health agent would be. Therefore, from a holistic perspective, decisive measures to *prevent* the next pandemic should receive considerable attention in the future (75). Future work could analyze medico-scientific contributions, field experiences, and role model aspects in more detail. The awareness of military's potential of threat and intimidation is crucial in order to prevent abuse. As success of disaster management in COVID-19 had a very strong local, tactical component, a thorough analysis of lessons learned from a micro-level CIMIC perspective may be informative to further strengthen cities and communities.

## Data availability statement

The original contributions presented in the study are included in the article, further inquiries can be directed to the corresponding author.

## Author contributions

MR conceived and designed this work, analyzed the data, wrote, and revised the manuscript and approved the submission. This work is part of MR's thesis project for a Master of Arts in Civil-Military Interaction at the Helmut Schmidt University/University of the Federal Armed Forces Hamburg, Germany.

## Funding

I acknowledge financial support by Deutsche Forschungsgemeinschaft within the funding program Open Access Publikationskosten as well as by Heidelberg University.

## Conflict of interest

The author declares that the research was conducted in the absence of any commercial or financial relationships that could be construed as a potential conflict of interest.

## Publisher's note

All claims expressed in this article are solely those of the authors and do not necessarily represent those of their affiliated organizations, or those of the publisher, the editors and the reviewers. Any product that may be evaluated in this article, or claim that may be made by its manufacturer, is not guaranteed or endorsed by the publisher.

## Author disclaimer

The views and opinions expressed in this publication are those of the author and do not necessarily reflect those of the affiliated institutions.
